# Investigating adherence to tyrosine kinase inhibitors in renal cancer

**DOI:** 10.1177/10781552241259354

**Published:** 2024-06-05

**Authors:** Fiona Angus, Yubo Wang, Alexander Rigg, Li-Chia Chen

**Affiliations:** 1 5294 Pharmacy Department, Christie NHS Foundation Trust, Manchester, UK; 2 Centre for Pharmacoepidemiology and Drug Safety, Division of Pharmacy and Optometry, School of Health Sciences, Faculty of Biology, Medicine and Health, 5292 University of Manchester, Manchester Academic Health Science Centre, Manchester, UK

**Keywords:** Renal cancer, tyrosine kinase inhibitors, adherence, persistency, medicines possession ratio

## Abstract

**Introduction:**

Tyrosine kinase inhibitors (TKIs) have been used as the first-line treatment for many patients with renal cell carcinoma (RCC), the seventh most common cancer in the United Kingdom. However, suboptimal adherence to TKIs can result in poor clinical prognosis. This study quantified RCC patients’ adherence to TKIs and explored factors associated with suboptimal adherence.

**Method:**

This retrospective cohort study was conducted at a specialist oncology tertiary hospital in Northwest England, using pharmacy dispensing records between November 2021 and March 2022. TKI prescriptions dispensed to patients with RCC were extracted to calculate the persistency gaps (≥7 or ≥14 days) and medication possession ratio (MPR). Multilevel regression analysis was conducted to associate MPR and persistency gaps with specific patient-related and TKI-related factors. This study did not require ethics approval.

**Results:**

Of the 2225 prescriptions dispensed to 109 patients, 469 (23.4%) and 274 (13.7%) persistency gaps of ≥7 and ≥14 days were identified. About 75% and 92% of patients had a persistency gap of ≥7 days within the first 90 days and 180 days. The length of time since the first TKI prescription (*p* < 0.001) and the use of sunitinib(*p* = 0.003) were significantly associated with the number of prescription gaps of ≥7 days. Moreover, the median MPR was 95.6% (interquartile range: 90.7%, 100.1%). Similarly, the length of time since the first TKI prescription was dispensed (*p* < 0.001) and the use of sunitinib (*p* = 0.034) were significantly associated with MPR.

**Discussion and conclusion:**

This single-centre study found that patients with RCC generally adhere to TKIs (MPR > 90%), but many patients experienced a persistency gap. The crucial window to mitigate TKI utilisation is within 180 days after the initial dispensing of TKIs. Further large-scale studies are required to comprehensively investigate other factors associated with adherence to TKIs and develop interventions to improve adherence and medication use problems.

## Introduction

Renal cancer is the 7^th^ most common cancer in the UK, with 13,300 cases diagnosed yearly.^1^ Most people are diagnosed in their 60 s and 70 s, with peak diagnosis at 75 years old.^2^ Risk factors for renal cancer include smoking, being overweight, age and hypertension.^2^ About 86% of renal cancers are categorised as renal cell carcinoma (RCC), and other types include papillary and a rare form, sarcomatoid.^3^ Moreover, 25% of RCC patients are diagnosed with metastases, 19% at stage 3 and 23% with stage 4 cancer. The 10-year survival overall is 52%, but the survival for those diagnosed with stage 4 cancer is 10% at 5 years.^1,4^

Treatment in cancer has moved towards using targeted therapies where the agent is modelled explicitly on the mechanism of action of the particular cancer growth.^5^ Introducing tyrosine kinase inhibitors (TKIs) has dramatically improved the clinical outcome of patients with advanced RCC by increasing overall and progression-free survival.^6^ TKIs act at the vascular endothelial growth factor molecule, stopping angiogenesis and halting or reducing tumour growth.^7^ Currently, TKIs are used in treating RCC in combination with immunotherapy or as single-agent therapy.^8^ Although TKIs have improved progression-free survival, problems involving resistance^9^ and side effects are also associated. Side effects such as diarrhoea, fatigue, nausea, mucositis and skin toxicities can affect adherence to TKI medication, as patients can find these intolerable. These can lead to the responsible clinician recommending that the patient pause treatment, reduce the dose or even discontinue the TKI despite having supportive medicines to counteract these side effects, such as anaesthetic mouthwash to help with mucositis.^10,11^

Medication adherence is ‘*the degree to which a person's behaviour agrees with the recommendations from a healthcare provider*’.^12^ Non-adherence is not a patient-driven problem but a multifactorial one involving the patient, therapy, health system, socioeconomics and the condition.^12,13^ It is not a new problem and can be seen as normal behaviour, as perfect adherence is unrealistic. However, with the advanced pharmaceutical development in oncology to extend the life expectancy of uncured cancer, suboptimal adherence to long-term treatment raises concerns about detrimental survival and quality of life.^14^ There is limited literature investigating the adherence to TKIs in patients with RCC; instead, increasing research has been undertaken on adherence to TKIs in chronic myeloid leukaemia (CML).^15^

CML is a rare disorder of bone marrow stem cells, with a low prevalence of 1.3 people per 100,000. It is generally diagnosed in the middle to old age with more males than females; 90% are diagnosed in the chronic stage, and 90% of CML patients will survive over 5 years.^16^ Adherence to TKIs in CML patients is reported as between 19% and 100%, depending on the adherence measure.^17–19^ Various measurements are available to quantify adherence, including direct (e.g. measuring drug level in blood) and indirect measures. Indirect measures include persistency gaps, medication possession ratio (MPR) and self-reporting measures, such as MMAS-8.^20^ However, no consensus exists on the gold standard measure for adherence.^20,21^ Indirect measurements, such as persistency gaps or MPR, measuring adherence information based on dispensing data, are considered objective, simple and easy to obtain.^21^

Compared to patients with CML, RCC is more prevalent, more patients are diagnosed later and older, and life expectancy is shorter. Prevalence and the factors influencing the non-adherence to TKIs (e.g. patients’ demography, disease severity, health literacy and self-efficacy) may differ between patients with CML and RCC. However, adherence to TKI therapy in RCC is an under-researched area. Therefore, this study evaluated the adherence to TKIs in RCC using a single hospital's dispensing records to explore the prevalence of suboptimal adherence and potential associated factors.^18^

## Method

### Study design and setting

This service evaluation was conducted at a tertiary cancer hospital in Northwest England. The data collection period was between November 2021 and March 2022. The research setting is a specialist oncology tertiary hospital that treated over 60,000 patients from April 2021 to March 2022 and serves a population of 3.2 million.^22^

The data collection was temporarily suspended between 21 December 2021 and 17 February 2022 due to restrictions related to the surge of COVID-19 infections in the region. Temporary suspension of much healthcare research was globally observed at this time.^23^ In this study, the suspension resulted in the re-organisation of the researcher's workload to collect and analyse all the data in a shorter period. However, it has a minimal impact on this study as all prescriptions of the study cohort during the follow-up period were retrospectively collected and analysed.

According to the NHS Health Research Authority's decision tool, ethical approval was not required for this service evaluation.^24^

### Study cohort and selection

The study cohort obtained were adult patients (age over 18 years) with RCC who were enrolled in the hospital's cyclical dispensing scheme (CDS) and dispensed with any TKIs, including axitinib, pazopanib, sunitinib, cabozantinib, tivozanib and lenvatinib from 2020 to 2021. Patients who received immunotherapy concurrently with TKI treatment or a compassionate supply of TKI were excluded from this study as having a compassionate or combination treatment may affect the motivation for taking TKI medication.

The CDS was established to reduce medication waste as systematic anti-cancer drugs (SACTs), including TKIs, tend to be expensive. When patients begin SACT treatment, their consultations are more frequent, which may help with adherence in the early phase of treatment. Stable patients who settled on SACT therapy can be accepted onto the CDS. Prescriptions issued at the clinic appointment usually can be dispensed monthly three times (cycles), and patients only need to meet their consultants/clinicians every 3 months to monitor treatment and side effects.

Following the CDS, led by a pharmacy team, the remainder of the medication will be posted in instalments until the next clinic appointment. An instalment is typically a cycle of medication. Patients are contacted one week before their next instalment is due, and as long as the patient is well and has not been told to stop treatment, the next instalment will be delivered to the patient. One of the advantages of this scheme includes a sooner referral to the oncology team if side effects are detected from treatment by the pharmacy team.^25^

The study cohort was followed from their first TKI prescription to the final day of the most recently dispensed prescription, and all TKIs dispensed to patients during the patient follow-up period were collected.

### Data collection

The patients’ year and month of birth, gender and information on their TKI prescriptions were collected from the hospital's JAC dispensing system. The date of birth was not recorded to maintain the patients’ anonymity. Month and year were documented for each patient, and birth was assumed to be the first day of the month. All information on TKIs dispensed for individual patients was collected from the first date of the first TKI dispensed to the patients until cessation due to a change of treatment or conclusion of SACT treatment. Drug name, prescribed dose, date of prescription and supply days of each dispensed TKI prescription were retrieved.

The data was collected using a pre-designed Microsoft Access form. A pseudo-number system was created to ensure no patient-identifiable information was up-loaded or taken off-site. Sequential numbers from one onwards were used for each patient and linked to their patient's hospital number. These numbers were on a spreadsheet kept on an encrypted file on a hospital password-secured laptop on site. A pilot study was carried out to explore any changes needed to revise the collection form, but no changes were required. Consequently, the data from the pilot study was incorporated into the study results.

### Outcome measures

The primary outcome was the MPR, which was used as a proxy to investigate patients’ adherence to TKIs, defined as ‘the proportion of a period where a medication supply is available’.^26^ In this study, this continuous variable was derived by dividing the sum of days of supply from each prescription by the total days of supply in the follow-up duration.^27^ Previous studies have used it to investigate adherence to TKIs in patients with CML.^28^ This study calculated MPR to reflect the assumption that all dispensed TKIs had been taken. Follow-up duration is the period between the date of the first prescription and the date of the last prescription covered. The formula used to calculate the MPR was as follows^29^:

$$\;\mathrm{MPR}=\left(\frac{\sum_{i=0}^{\mathrm{end}\;\mathrm{of}\;\mathrm{the}\;\mathrm{interval}\;\mathrm{period}}\;\mathrm{Days}\;\mathrm{supply}\;\mathrm{of}\;Rx_{i}}{\mathrm{Days}\;\mathrm{of}\;\mathrm{interval}\;\mathrm{period}}\right)\times 100=percentage\;(\text{\%})$$

Typically, the results of MPR are expressed as a percentage; an MPR value of 0% indicates no adherence, and 100% represents theoretically perfect adherence. Moreover, if MPR were recorded above 100%, patients would have collected more medication than required to achieve perfect adherence.^5^ Besides, this study explored the proportion of patients with MPR < 80% or MPR < 90%, which is conventionally used to define suboptimal adherence in cancer^30^ or other chronic disease conditions.^31–35^

The secondary outcome was persistency gaps, which showed periods of suboptimal adherence that could be identified. It was assumed all dispensed medication was consumed and none was discarded or expired. Persistency gaps were calculated by having a running total of dispensed medication and identifying gaps of more than 7 or 14 days from the end of the first prescription to the beginning of the last prescription.

### Data analysis

Descriptive statistics were used to demonstrate patient characteristics: age, gender, prescribed TKI and length of follow-up. Continuous outcomes were recorded as mean, standard deviation, median and interquartile range (IQR).

Two linear regression models (univariate and multilevel regression analyses) were carried out on the two outcome measures of adherence, i.e. MPR and persistency gaps, to examine the effect of single and multiple variables on the dependent adherence variable. The independent variables of both models included age, gender, types of TKI and follow-up period. Male patients and patients taking cabozantinib were used as baselines to compare against female patients and patients taking the other TKIs. Coefficient and 95% confidence intervals (95%CI) for each independent variable of the regression model were reported, and a *p*-value less than 0.05 was considered statistically significant.

Finally, time-to-event analysis was used to analyse the probability of the events of interest. In this case, the event was the first persistency gap of over 7 days and 14 days over the follow-up time. Data was collected using Microsoft Access version 2204 (Microsoft Corporation 2022), and analysis was performed using Microsoft Excel version 2204 (Microsoft Corporation 2022) and Stata 14 (StataCorp. 2015. Stata Statistical Software: Release 14).

## Results

### Characteristics of participants

**Figure 1.**
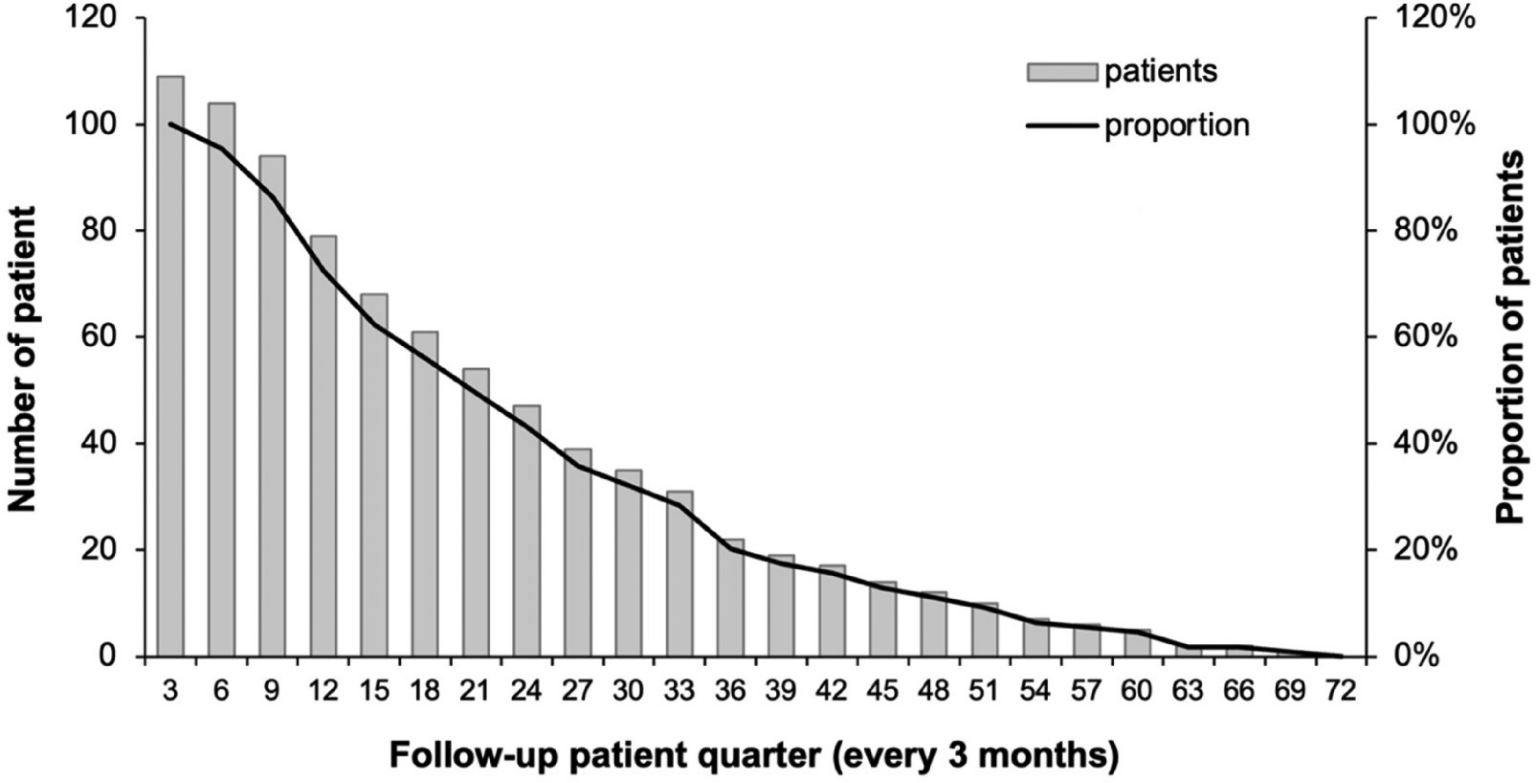
Number of patients in each follow-up patient quarter.

During the study period, 2225 prescriptions for dispensed TKIs from 109 patients were collected from the JAC dispensing system. All 109 patients were prescribed TKIs to treat metastatic/advanced RCC and were on the CDS. The median age of the patient sample was 68 years old, with an IQR of 60–74 years. 64.8% (n = 51) of the patients were over 70 years old, with the oldest patient being 88 years old. The proportion of males (n = 66; 60.1%) was higher than females (n = 43; 39.4%). The patients’ follow-up period ranges from 78 to 2103 days, with a mean of 975.5 ± 518.1 days (2.6 ± 1.4 years). About 56% of patients had more than or equal to 15 months of follow-up periods (Figure 1).

The mean number of prescriptions dispensed per patient was 19.4 ± 11.8 prescriptions. The most frequently prescribed TKI was cabozantinib (n = 48), after which it was tivozanib (n = 22), followed by sunitinib (n = 21), pazopanib (n = 10), axitinib (n = 5) and lenvatinib (n = 3). The most frequently prescribed TKI categories are based on the patient's most recently prescribed TKIs. For example, if a patient started his/her TKI treatment from cabozantinib but is now using sunitinib, they will be included in the sunitinib category.

Thirty-seven out of the 109 patients had their prescribed dose of TKI reduced during the follow-up period. The dose reduction occurred while patients were prescribed cabozantinib (n = 21), tivozanib (n = 7), sunitinib (n = 5), axitinib (n = 2) and pazopanib (n = 2), and no patients taking lenvatinib had dose reductions during the follow-up period.

### Medication possession ratio

**Figure 2.**
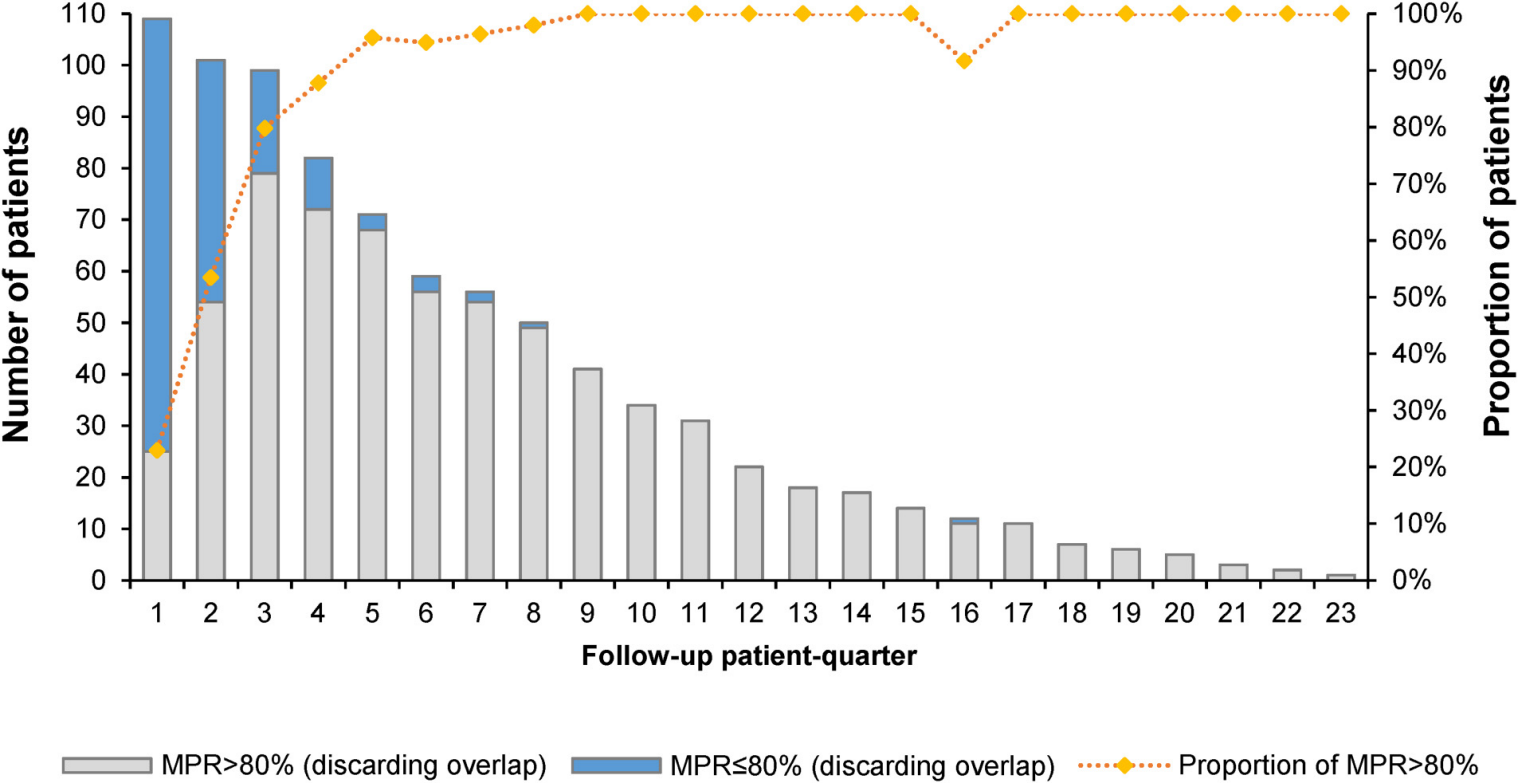
Number and proportion of patients with MPR over 80% per follow-up patient quarter. MPR: medication possession ratio.

**Figure 3.**
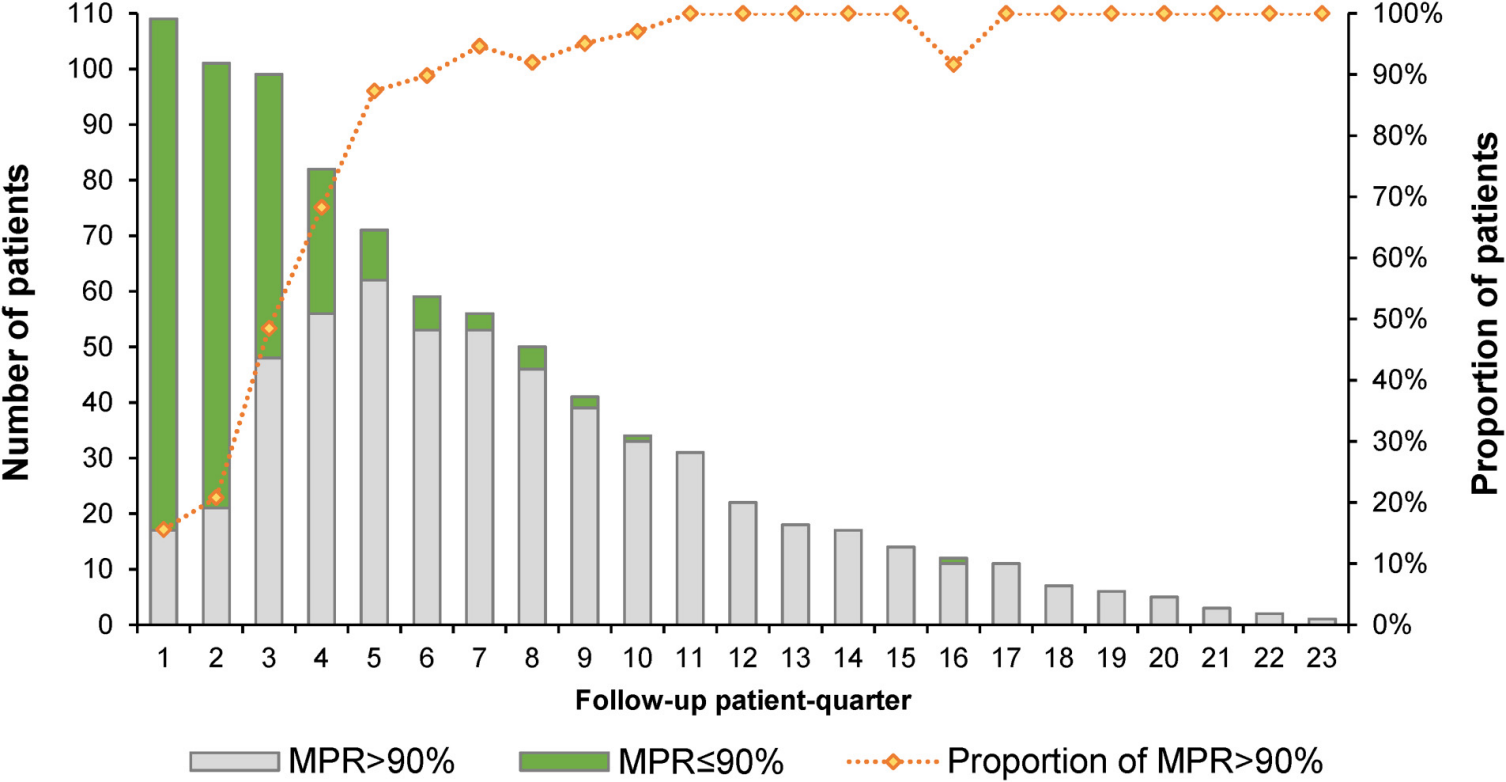
Number and proportion of patients with MPR over 90% per follow-up patient quarter. MPR: medication possession ratio.

The median MPR was 95.6% (IQR: 90.7%, 100.1%), ranging from 61.5% to 141.2%. Of the 109 patients, 78% had an MPR of over 90%, and 95.4% had an MPR of over 80%. Following the proportion of patients with MPR over 80% (Figure 2) or 90% (Figure 3) in each patient quarter, most suboptimal adherence (MPR < 80% or MPR < 90%) occurred in the first 12 months following the first prescription. After 2 years of receiving the same TKI, almost all patients showed optimal adherence for the remainder of the time they took the TKI.

The univariate regression analysis, which analyses a single dependent and predictor variable, showed that, except for the type of TKIs and follow-up duration, other factors were not significantly associated with MPR. Compared to all other TKIs against cabozantinib, only the use of sunitinib significantly increased MPR (coefficient 3.81%; 95%CI: 0.07%, 7.56%; *p* = 0.046). Increasing one follow-up day significantly increased 0.0093% (95%CI: 0.0070%, 0.0116%; *p* < 0.001) MPR (Appendix 1).

Likewise, the multilevel regression analysis considered all variables and their correlation and found that using sunitinib (compared against cabozantinib and the length of follow-up) was also significantly associated with MPR (R-square value 0.4673). However, compared to all other TKIs against cabozantinib, only the use of sunitinib significantly reduced MPR (coefficient −3.68%; 95%CI: −7.09%, −0.28%; *p* = 0.034). This shows increasing one follow-up day significantly increased 0.0119% (95%CI: 0.0092%, 0.0146%; *p* < 0.001) MPR (Appendix 1).

### Persistency gaps between prescriptions

**Figure 4.**
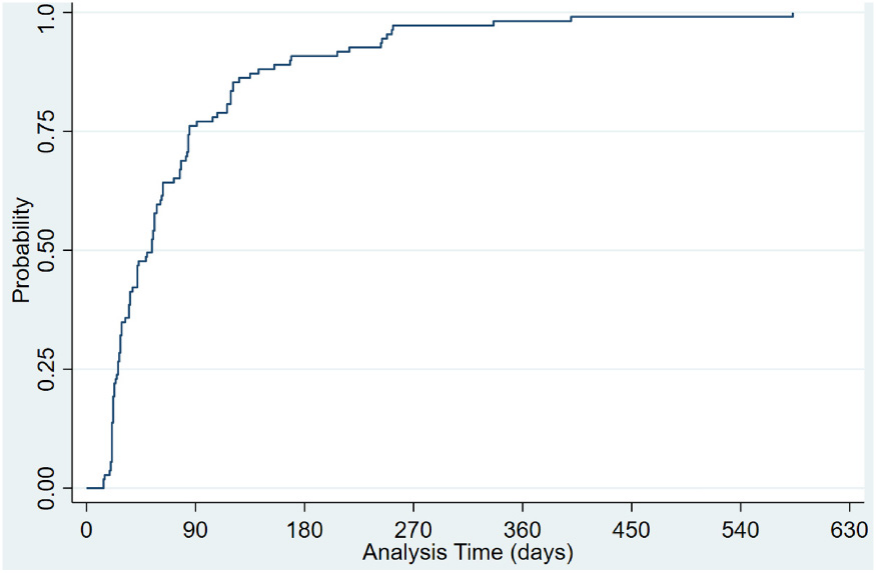
Time-varying probability of patients with a persistency gap of equal or more than 7 days. Probability: The proportion of patients with a persistency gap of equal or more than 7 days. Analysis of days: Time (days) to the first persistency gap of more than or equal to 7 days.

**Figure 5.**
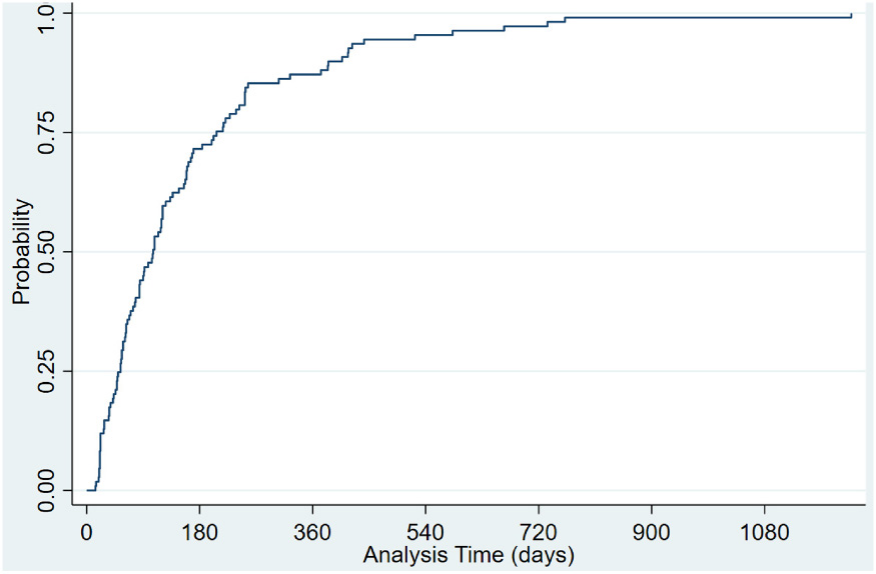
Time-varying probability of patients with a persistency gap of equal or more than 14 days. Probability: The proportion of patients with a persistency gap of equal or more than 14 days. Analysis of days: Time (days) to the first persistency gap of more than or equal to 14 days.

Of the 2114 gaps between dispensed prescriptions, 469 (23.4%) were persistency gaps of 7 days or more, and 274 (13.7%) were persistency gaps of 14 days or more. For most patients with a persistency gap of 7 days or more, the first persistency gap (≥7 days) was within the first 180 days following their first TKI prescription (Figure 4), and 75% of patients had this gap within the first 90 days. Comparably, 70% of patients have a persistency gap of more than 14 days within the first 180 days following their first TKI prescription. However, the remaining patients took much longer to have a gap of more than 14 days than when a persistency gap of 7 days or more was defined (Figure 5). This shows that the early stages of taking TKIs are the critical time for suboptimal adherence.

The univariate analysis found that female patients (*p* = 0.032) and the length of time since the first TKI prescription (*p* < 0.001) were significantly associated with the increased number of prescription gaps of 7 days or more (Appendix 2). However, only the time since the first TKI prescription was significantly associated with the prescription gap of more than 14 days (*p* < 0.001) (Appendix 3). Without considering the correlation between other covariates, the longer the time that patients receive, the more persistency gaps are observed.

In contrast, multilevel regression analysis found that the use of sunitinib (*p* = 0.003) and the length of time since the first TKI prescription (*p* < 0.001) were significantly associated with the number of prescription gaps of 7 days or more (R-square: 0.5357) (Appendix 2). Similarly, both sunitinib (*p* = 0.001) and the length of time (*p* < 0.001) were significantly associated with the number of prescription gaps of more than 14 days (R-square: 0.4127). This result showed the correlation between covariates and clustering effect, such as the type of TKIs.

## Discussion

This study assessed adherence to TKI medication in advanced RCC patients using single-centre hospital records. Two adherence measures (MPR and persistence gap) were employed. Suboptimal adherence was defined through subgroup analysis, considering different MPR cut-offs and persistency gap thresholds. Factors associated with suboptimal adherence were explored using regression analysis to identify potential predictive factors and their correlation.

Although the literature has proved TKIs’ efficacy in improving the survival of patients with RCC,^6^ common TKI-related adverse effects may jeopardise patients’ quality of life, and rare and serious adverse effects could lead to poor consequences requiring hospitalisation.^36,37^ Moreover, a better insight into factors predicting adverse effects and suboptimal adherence can help optimise the individualised treatment regimen and ensure a better quality of life by avoiding the adverse effects.

Overall, this study found that most patients with RCC who received TKIs from the CDS had an optimal adherence to TKIs based on the MPR measure. The median MPR was 95.6%, and over three-quarters (78%) of patients had an MPR greater than 90%. Despite no standardised measure for adherence and no agreed cut-off of MPR to classify adherence levels, MPR > 90% has been used in previous studies considering adherence to TKIs in patients with CML.^28,38–40^ Although there is currently limited research on the utilisation of TKIs in patients with RCC, referring to other studies on TKIs in cancer treatment, these aggregate level results suggest that patients with RCC had a reasonable adherence to TKIs.

Similar to this study, the previous literature reported the median MPR for adherence to TKIs in patients with CML ranging from 95% (IQR: 83%, 107%),^38^ 98.3% (Range: 12.6%, 100%)^39^ to 99% (Range: < 0.01%, 203%).^40^ In patients with CML, the proportion of patients with MPR > 90% ranged from 69.4%^38^ to 73.1%^39^ for TKIs, 86% for nilotinib and 74% for imatinib.^28^ Besides, patients with optimal adherence to TKIs, defined by MPR > 90%, have also been associated with improving survival in a retrospective cohort study of 2870 CML patients diagnosed between 2005 and 2013 in Korea.^40^ However, further research is warranted to assess whether high-level adherence to TKIs improves survival in patients with RCC.

Furthermore, this study investigated the persistency gaps in patients taking TKI medication, which was under-studied in published research. This study found 23.4% and 13.7% of patients had persistency gaps of 7 days or 14 days or more, respectively. Notably, these gaps are commonly found during the early stage of TKI treatment. It is plausible that these early-stage gaps indicated intentional non-adherence attributed to either patient-self-managed or clinician-recommended breaks due to side effects of TKI therapy.

This study employed a preliminary analysis of MPR and persistency gaps by applying relevant variables available in the electronic medical records and found the duration since the initial prescription of TKI medication was the most significant factor in adherence. The crucial duration was within the first 12 months of taking TKIs, as over 75% of patients had a persistency gap of 7 days or more in the first 9 months, and all patients had one in the first year. Moreover, the quarterly MPR measure also found that in the first quarter of year 2, 87.3% of patients had an MPR > 90%, which was significantly higher than any quarter in year 1. Therefore, adherence to TKIs in patients with RCC should be closely monitored during the first 12 months of taking TKIs, and potential interventions may also be implemented in this period.

Cabozantinib was the most frequently prescribed TKI, accounting for the largest proportion of dose changes. It served as the reference when assessing the impact of different TKIs on MPR and the occurrence of persistency gaps. Among the five TKIs compared with cabozantinib, sunitinib was the only TKI significantly associated with a reduction in both MPR and persistency gaps, as revealed in multilevel regression analyses. This counterintuitive impact cannot be solely explained by sunitinib's known adverse effects,^41,42^ given the presence of unmeasured confounding factors^43^ and the relatively small number of patients prescribed sunitinib. Future studies should explore how patient-related, healthcare provider-related and healthcare system-related factors influence adherence to oral anti-neoplastic agents.^44^ Univariate regression showed a positive effect of sunitinib on MPR, but as multilevel regression demonstrated a negative effect, this again emphasises unmeasured confounding factors influencing this result.

Indeed, various interventions were implemented at the research site of this study to enhance patients’ adherence to oral systemic anti-cancer treatment. These interventions included (i) an around-the-clock hotline for urgent queries, (ii) specialist nurses available to address telephone queries regarding side effects and medication administration, and (iii) the CDS, where monthly interactions with a pharmacy technician facilitate repeat dispensing. During these interactions, the pharmacy technician explored questions about adherence and advised patients to contact the renal cancer team, constituting an intervention to promote adherence. It's essential to note that the nature and implementation of these adherence aids may vary across cancer centres. Therefore, caution is warranted when generalising optimal adherence rates to patients with RCC treated in different centres.

This single-centre cohort study used hospital medication records to explore patients’ adherence to TKIs using two different measures (i.e. the MPR and persistence gap). Subgroup analysis was adopted to explore the impacts of definitions, i.e. the MPR cut-off for optimal adherence and the persistency gap measure as 7 or 14 days or more. Also, a regression analysis was applied to relevant variables to identify potential predictive factors.

Limitations impede the generalisability of the results from this study, such as a single-centre study with limited sample size and follow-up duration. Besides, the disease conditions and treatment regimens of patients enrolled in the CDS were deemed stable to receive repeat dispensing. Hence, patients with a high risk of suboptimal adherence were omitted. Furthermore, it is essential to recognise that clinicians may recommend some treatment breaks leading to persistence gaps. These breaks are unrelated to intentional patient non-adherence, which is generally unknown to clinicians. Therefore, further research is required to reduce the need for clinician-led treatment breaks by optimising patient support.

Moreover, the MPR was a proxy to measure adherence. Lam and Fresco indicated that MPR is a simple calculation that does not consider gaps between prescriptions, so the value obtained can be overestimated.^45^ Besides, the calculation assumption that ‘patients took all dispensed medications as prescribed’ needs further validation. To test the impact of this assumption, this study also calculated MPR by discarding any ‘leftover’ of dispensed TKIs before the next prescription was dispensed (MPR2). Under this assumption, the median MPR2 was 93.8% (IQR: 84%, 96.8%), and 76.2% and 91.7% of patients had an MPR2 > 90% and 80%, respectively. These results did not change the conclusion.

To improve adherence to TKIs in patients with RCC, research is needed to explore the reasons for suboptimal adherence further by retrospectively exploring patients’ medical records or conducting interviews with patients. By incorporating patients’ perspectives and care needs, it is possible to scope the support and interventions for improving medication adherence.^46^ Besides, as medication adherence is a multi-faced issue, patients’ health literacy,^47^ self-efficacy^48^ and health equality issues warrant further investigation. Moreover, it is also essential to evaluate the relationship between medication adherence, treatment efficacy and the ultimate outcomes of treatments, i.e. overall survival and quality of life in patients with RCC.

## Declaration

### Consent for publication

This study contains original, unpublished work and is not being submitted for publication elsewhere at the same time. Parts of the results were reported as poster presentations at the following conferences:

FA, YW, AR and LCC. Investigating adherence to TKIs in patients with renal cancer. Poster presentation. British Oncology Pharmacy Association 25th Annual Symposium, 7–9 October 2022, ACC Liverpool, UK.

FA, YW, AR and LCC. Investigating dispensing persistency of TKIs for patients with renal cancer. Poster presentation. UK Clinical Pharmacy Association Virtual Conference 2022, 10 November 2022.

## Appendices

**Appendix 1.** Factors associated with the medicines possession ratio of tyrosine kinase inhibitors.

Covariate	Co-efficient (95%CI)	*p*-value
Univariate regression analysis		
Age (year)	0.0010076 (−0.0004246, 0.0024398)	0.166
Female (compared with male)	0.009901 (−0.0184938, 0.0382957)	0.491
*Compared with cabozantinib*		
Tivozanib	0.0326622 (−0.0041866, 0.069511)	0.082
Sunitinib	0.0381327 (0.0006871, 0.0755783)	0.046*
Pazopanib	0.0430258 (−0.0067249, 0.0927766)	0.089
Axitinib	−0.0050072 (−0.0722644, 0.06225)	0.883
Lenvatinib	0.0209906 (−0.064184, 0.1061652)	0.626
Length of time since first TKI prescription	0.000093 (0.0000702, 0.0001159)	<0.001*
Multilevel regression analysis		
Age (year)	−0.0003851 (−0.0015884, 0.0008182)	0.527
Female (compared with male)	−0.0201047 (−0.0429361, 0.0027266)	0.084
*Compared with cabozantinib*		
Tivozanib	0.0277411 (−0.0023623, 0.0578445)	0.070
Sunitinib	−0.0368406 (−0.0708782, −0.0028031)	0.034*
Pazopanib	0.0025439 (−0.0375985, 0.0426864)	0.900
Axitinib	−0.0416045 (−0.0940455, 0.0108364)	0.119
Lenvatinib	0.0224051 (−0.0435704, 0.0883806)	0.502
Length of time since first TKI prescription	0.000119 (0.0000916, 0.0001463)	0.001*

(Note) 95%CI: 95% confidence interval; *significant difference. Male patients and patients taking cabozantinib were used as the reference.

**Appendix 2.** Factors associated with the number of prescription gaps equal to or more than 7 days.

Covariate	Co-efficient (95%CI)	*p*-value
Univariate regression analysis		
Age (year)	0.0185699 (−0.0538933, 0.0910331)	0.612
Female (compared with male)	1.535588 (0.1375346, 2.933642)	0.032*
*Compared with cabozantinib*		
Tivozanib	−0.6193182 (−2.470778, 1.232141)	0.509
Sunitinib	1.824405 (−0.0570399, 3.705849)	0.057
Pazopanib	1.3625 (−1.137215, 3.862215)	0.282
Axitinib	1.0625 (−2.316821, 4.441821)	0.534
Lenvatinib	−1.270833 (−5.550412, 3.008745)	0.557
Length of time since first TKI prescription	0.0052714 (0.0042257, 0.0063171)	<0.001*
Multilevel regression analysis		
Age (age)	−0.0252821 (−0.081683, 0.0311189)	0.376
Female (compared with male)	0.2168039 (−0.8533216, 1.286929)	0.689
*Compared with cabozantinib*		
Tivozanib	−1.074332 (−2.485304, 0.3366398)	0.134
Sunitinib	−2.430616 (−4.025986, −0.8352459)	0.003*
Pazopanib	−1.171579 (−3.05309, 0.7099314)	0.220
Axitinib	−1.044362 (−3.502314, 1.41359)	0.401
Lenvatinib	−1.315709 (−4.408037, 1.776619)	0.401
Length of time since first TKI prescription	0.0062724 (0.0049904, 0.0075544)	<0.001*

(Note) 95%CI: 95% confidence interval; *significant difference. Male patients and patients taking cabozantinib were used as the reference.

**Appendix 3.** Factors associated with the number of prescription gaps equal to or more than 14 days.

Covariate	Co-efficient (95%CI)	*p*-value
Univariate regression analysis		
Age (year)	0.006683 (−0.0416713, 0.0550374)	0.785
Female (compared with male)	0.879845 (−0.0576577, 1.817348)	0.066
*Compared with cabozantinib*		
Tivozanib	−0.5909091 (−1.845544, 0.6637258)	0.352
Sunitinib	0.452381 (−0.8225733, 1.727335)	0.483
Pazopanib	0.6605556 (−0.9939231, 2.393923)	0.414
Axitinib	0.2210866 (−1.989985, 2.589985)	0.796
Lenvatinib	−1.166667 (−4.066708, 1.733374)	0.427
Length of time since first TKI prescription	0.0029053 (0.0021117, 0.0036988)	<0.001*
Multilevel regression analysis		
Age (age)	−0.0165851 (−0.0588748, 0.0257046)	0.438
Female (compared with male)	0.1785503 (−0.6238346, 0.9809352)	0.660
*Compared with cabozantinib*		
Tivozanib	−0.8593202 (−1.917273, 0.1986331)	0.110
Sunitinib	−2.090057 (−3.286273, −0.8938412)	0.001*
Pazopanib	−0.8204768 (−2.231242, 0.5902884)	0.251
Axitinib	−0.9659882 (−2.808972, 0.8769953)	0.301
Lenvatinib	−1.208565 (−3.527207, 1.110077)	0.304
Length of time since first TKI prescription	0.0037385 (0.0027772, 0.0046998)	<0.001*

(Note) 95%CI: 95% confidence interval; *significant difference. Male patients and patients taking cabozantinib were used as the reference.
